# Hierarchically Assembled Plasmonic Metal-Dielectric-Metal Hybrid Nano-Architectures for High-Sensitivity SERS Detection

**DOI:** 10.3390/nano12030401

**Published:** 2022-01-26

**Authors:** Puran Pandey, Min-Kyu Seo, Ki Hoon Shin, Young-Woo Lee, Jung Inn Sohn

**Affiliations:** 1 Division of Physics and Semiconductor Science, Dongguk University-Seoul, Seoul 04620, Korea; ppcpurans@gmail.com (P.P.); seominkyuu@gmail.com (M.-K.S.); kihoonshin@dongguk.edu (K.H.S.); 2 Department of Energy Systems, Soonchunhyang University, Asan-si 31538, Korea

**Keywords:** SERS, metal-dielectric-metal, Au nanoparticles, hot spots, FDTD simulation

## Abstract

In this work, we designed and prepared a hierarchically assembled 3D plasmonic metal-dielectric-metal (PMDM) hybrid nano-architecture for high-performance surface-enhanced Raman scattering (SERS) sensing. The fabrication of the PMDM hybrid nanostructure was achieved by the thermal evaporation of Au film followed by thermal dewetting and the atomic layer deposition (ALD) of the Al_2_O_3_ dielectric layer, which is crucial for creating numerous nanogaps between the core Au and the out-layered Au nanoparticles (NPs). The PMDM hybrid nanostructures exhibited strong SERS signals originating from highly enhanced electromagnetic (EM) hot spots at the 3 nm Al_2_O_3_ layer serving as the nanogap spacer, as confirmed by the finite-difference time-domain (FDTD) simulation. The PMDM SERS substrate achieved an outstanding SERS performance, including a high sensitivity (enhancement factor, EF of 1.3 × 10^8^ and low detection limit 10^−11^ M) and excellent reproducibility (relative standard deviation (RSD) < 7.5%) for rhodamine 6G (R6G). This study opens a promising route for constructing multilayered plasmonic structures with abundant EM hotspots for the highly sensitive, rapid, and reproducible detection of biomolecules.

## 1. Introduction

Owing to its extremely high sensitivity, ability to work in real time, and multiplexing detection capability, surface-enhanced Raman scattering (SERS) has emerged as a powerful detection technique for sensing molecules through its unique fingerprint vibrational spectrum [1,2,3,4,5]. It has tremendous potential for single-molecule level detection [6,7], the investigation of live cells [8,9], the monitoring of catalytic reactions [10,11], and sensing molecules, in both liquid and solid samples [12,13]. In SERS, the Raman signals of analytes can be amplified by several orders of magnitude (10^8^–10^10^) based on two mechanisms: electromagnetic mechanism (enhancement of ~10^6^–10^8^) and chemical mechanism (enhancement of ~10^2^–10^4^) [14,15,16]. The SERS enhancement mostly relies on the amplification of the electromagnetic field—i.e., electromagnetic (EM) hot spots generated by the excitation of the localized surface plasmon resonance (LSPR) of the metal nanostructures [17]. Therefore, plasmonic metallic nanostructures including Au, Ag, and Cu have been fabricated to prepare an excellent SERS substrate for sensing molecules [18,19,20]. Designing and optimizing the geometry of a plasmonic nanostructure, such as its size [21], sharp corners [22], tips [23], surface roughness [24], and interparticle gaps [25], is essential in order to provide a strong EM hotspot and hence enhance SERS signal intensities. Among these, the interparticle gap structure has attracted considerable attention thanks to its ability to provide extremely strong EM hot spots within a sub-nanometer gap [26]. The precise control of nanogaps between plasmonic nanoparticles (NPs) at a nanometer scale is crucial to produce a high density of strong and stable EM hot spots. To maintain the specific sub-nanometer gap, a dielectric layer can be considered as a nanogap spacer between two layered plasmonic metal nanostructures—namely, metal-dielectric-metal hybrid nano-architectures [27,28,29,30]. The dielectric spacer offers several benefits: protecting the plasmonic core from oxidation, tunning the LSPR properties, and maintaining a sub-nanometer gap between metal nanostructures to obtain a strong EM hotspot [31,32,33,34]. Therefore, it is of great significance to construct a unique 3D nano-architecture SERS substrate that comprises a hierarchical assembly of plasmonic NPs, separated by a dielectric spacer, for achieving an extremely high SERS activity.

Inspired by the above discussion, we report a facile method for fabricating abundant nanogaps containing hierarchically assembled 3D plasmonic metal-dielectric-metal (PMDM) hybrid nano-architectures for superior SERS detection in this work. However, the developed PMDM hybrid SERS sensor is considerably different from the above-mentioned metal-dielectric-metal structure in terms of the preparation method and architecture of the SERS platform. The PMDM hybrid nanostructures were prepared by the thermal evaporation of Au film followed by the thermal annealing and atomic layer deposition (ALD) of the Al_2_O_3_ dielectric layer. We achieved an enormous SERS enhancement of the PMDM hybrid nanostructures, with a maximum enhancement factor (EF) of 1.3 × 10^8^ and a low detection limit of 10^−11^ M R6G molecules. We further observed the excellent reproducibility of the SERS substrate with relative standard deviation (RSD) values of less than 7.5%. To support the experimental SERS performance, we conducted the finite-difference time-domain (FDTD) simulation of hybrid nanostructures and showed that a high density of strong EM hot spots was produced between the Au core and numerous out-layered Au NPs at the Al_2_O_3_ nanogap regions.

## 2. Materials and Methods

### 2.1. Hybrid Nanostructures Fabrication

SiO_2_/Si substrates (purchased from Sehyoung wafertech, Seoul, Korea) were cleaned by acetone, isopropyl alcohol (Sigma-Aldrich, Saint Louis, MO, USA), and deionized water for 10 mins in sequence in an ultrasonic cleaner and dried at room temperature. Au films (10 nm) were deposited on SiO_2_/Si substrates using thermal evaporation, where the thickness of films was controlled by the deposition time. The 10 nm Au films was annealed in a rapid thermal annealing (RTA) chamber at 800 °C for 120 s for the fabrication of core Au NPs arrays based on the solid-state dewetting [35,36]. Subsequently, the ultrathin dielectric layer—i.e., 3 nm Al_2_O_3_ film—was deposited on the as-prepared core Au NPs via the ALD method. Then, the 5nm Au films were deposited on the surface of Au/Al_2_O_3_ nanostructures, followed by annealing at 450 °C for 120 s to produce highly dense small-sized Au NPs on the surface of Au/Al_2_O_3_ NPs, which are referred to as a hierarchically assembled PMDM (Au/Al_2_O_3_/Au) nanostructures. For comparison, double dewetting Au nanostructures were prepared by a similar method without using an Al_2_O_3_ spacer.

### 2.2. Sample Characterization

The morphological characterization and elemental analysis of the as-synthesized hybrid nanostructures fabricated on SiO_2_/Si substrates was performed using a field emission scanning electron microscope (FE-SEM, Hitachi S-7400, Tokyo, Japan), coupled with the energy-dispersive x-ray spectroscopy (EDS) analysis. Moreover, the crystalline information was examined by X-ray diffraction (XRD, Rigaku Ultima IV diffractometer, Tokyo, Japan) with Cu-Kα radiation, whereas the chemical states were evaluated using X-ray photoelectron spectroscopy (XPS, Veresprobe II, Ulvac-phi, Chigasaki, Japan).

### 2.3. SERS Analysis

Rhodamine 6G (R6G, Sigma-Aldrich, Saint Louis, MO, USA) was used as a probe molecule to determine the SERS activity of the as-prepared PMDM hybrid nanostructures. For the preparation of the samples for SERS measurements, the SERS substrates were immersed in different concentrations of R6G solution ranging from 10^−12^ to 10^−5^ M for 2 h to allow the sufficient adsorption of R6G molecules on plasmonic nanostructures. SERS measurements were performed using confocal Raman spectroscopy (HEDA, NOST, Seongnam, Korea) at room temperature. SERS signals were acquired using an incident laser with a wavelength of 532 nm with a power of 0.1 mW (laser spot size ~1 μm), 100× objective lens (numerical aperture = 0.80), and acquisition time of 10 s. To determine the EF, the Raman spectrum of R6G (10^−2^ M) adsorbed on the SiO_2_/Si substrates was evaluated as above.

### 2.4. FDTD Simulation

The EM field distribution was calculated with the FDTD method (Lumerical Solutions Inc., Vancouver, BC, Canada). In our simplified unit of the simulation model, the core Au NP was assumed to be a larger hemisphere coated with a dielectric spacer layer (3 nm Al_2_O_3_) followed by the out-layered small Au NPs. The diameter of the core, top, and surrounding Au NPs was supposed to be 180, 60, and 30 nm, respectively. Furthermore, the incident light source of a plane wave, surrounding medium (air), perfectly matched layer (PML) as an absorption boundary in z-boundary, periodic boundary condition for x and y directions, and mesh size (1 nm) were selected for the simulation to compute the EM field distribution. The near-field EM field intensity was calculated in the vicinity of the nanostructures using two monitors in X-Y and Y-Z directions. The data of a refractive index for Au were obtained from the Johnson and Christy model [37]. The data for SiO_2_ and Al_2_O_3_ were acquired from the model data provided by the software.

## 3. Results and Discussion

**Figure 1:**
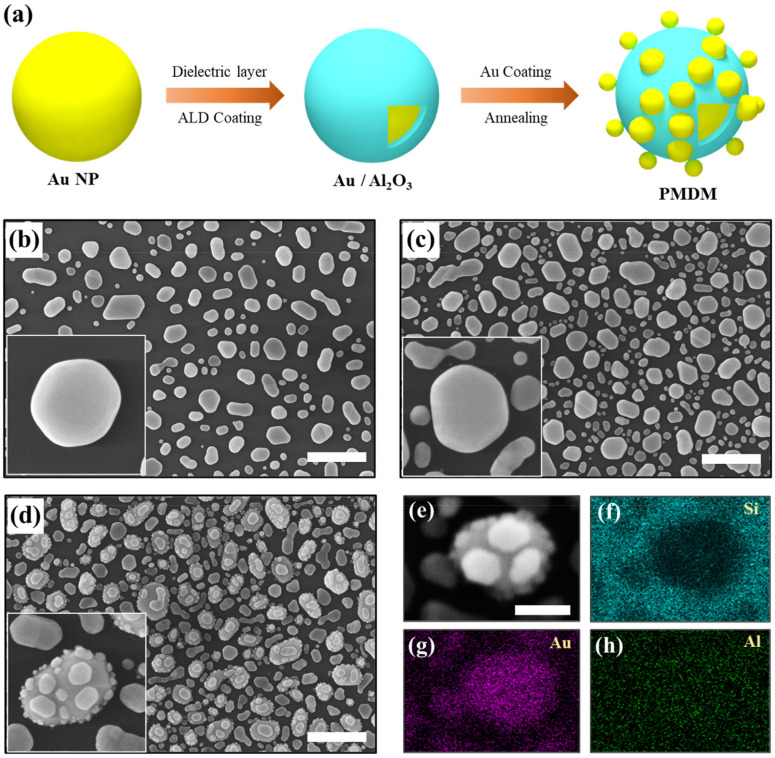
(**a**) Schematic illustration of the fabrication of hierarchically assembled plasmonic metal-dielectric-metal (PMDM) nano-architectures. (**b**) SEM image of Au nanoparticles (NPs) fabricated on Si/SiO_2_ substrate by the annealing of Au film (10 nm) at 800 °C for 120 s. (**c**) Fabrication of double dewetting Au NPs arrays grown on Au NPs (Au/Au) by the deposition of 5 nm Au film and subsequent annealing at 450 °C for 120 s. (**d**) SEM image of hierarchically assembled PMDM hybrid nano-architectures. Scale bar: 400 nm. (**e**–**h**) EDS mapping of PMDM hybrid nanostructures grown on Si/SiO_2_ substrate, where elemental maps are Si (blue), Au (red), and Al (green). Scale bar: 100 nm.

Figure 1a shows the fabrication procedures and surface morphology of the hierarchically assembled PMDM hybrid nanostructures fabricated on Si/SiO_2_ substrate. The combination of double dewetting and the ALD approach was employed for the preparation of the SERS substrates. The aim of using this approach with a dielectric layer between Au NPs is to obtain hierarchical nano-architectures with a strong plasmonic response and massive gap-introduced EM hot spots. First, high-density core Au NPs arrays were prepared based on the thermal dewetting of 10 nm Au thin film at 800 °C for 120 s. The average diameter Au NPs was found to be ~136 nm and the corresponding size distribution is shown in the histogram of Figure S1. The surface morphology of the fabricated Au NPs on the substrate with well-dispersed semispherical or somewhat faceted NPs is shown in the SEM image of Figure 1b. Next, we deposited 5 nm Au films on as-prepared core Au NPs arrays and then annealed them at 450 °C for 120 s. This repeated dewetting of Au films resulted in the formation of a very high density of Au NPs, as shown in Figure 1c. The larger core Au NPs were surrounded by comparatively small Au NPs. However, the spacing between them was too large, meaning they cannot be considered a good candidate for SERS substrates. Therefore, we deposited a 3 nm Al_2_O_3_ thin film on core Au NPs to fabricate a metal-dielectric core-shell nanostructure via the ALD approach. Subsequently, a 5 nm Au thin film was deposited on the Au/Al_2_O_3_ nanostructure followed by annealing at 450 °C for 120 s, which gives rise to the formation of hierarchically assembled PMDM hybrid nanostructures, as shown in Figure 1d. These hierarchical PMDM hybrid nanostructures provide not only an increased surface coverage and roughness, but also multiple out-layered small-sized Au NPs separated with a nanogap layer of dielectric Al_2_O_3_ from the core Au NPs. The out-layered Au NPs can be distinctly observed along the surface of core Au NPs in Figure 1d. Moreover, we confirmed the formation of PMDM hybrid nanostructures by using EDS mapping based on elemental analysis, as shown in Figure 1e–h.

**Figure 2:**
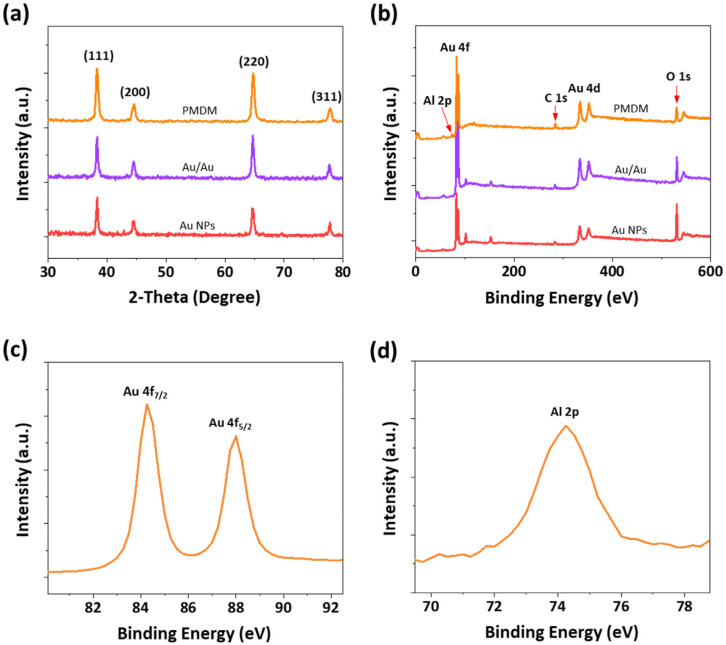
(**a**) XRD patterns of three different plasmonic nanostructures: Au, Au/Au, and PMDM hybrid nanostructures, as labeled. (**b**) XPS survey spectra of Au, Au/Au, and PMDM hybrid nanostructures. (**c**,**d**) High-resolution XPS spectra of Au 4f and Al 2p of PMDM hybrid nanostructure.

The crystalline structures of the Au and hybrid nanostructures were examined with an XRD pattern, in which all samples possessed almost the same diffraction peaks as those shown in Figure 2a. Four distinct diffraction peaks were observed at 38.3, 44.3, 64.7, and 77.7° corresponding to the (111), (200), (220), and (311) planes of the face-centered cubic phase of Au (JCPDS no. 04-784), revealing the formation of Au NPs. Furthermore, the XPS spectra of the Au and PMDM hybrid nanostructures were thoroughly analyzed to confirm the elemental and chemical states. Figure 2b shows the XPS survey spectra of the Au NPs, double dewetted Au/Au NPs, and PMDM hybrid nanostructures, discovering all the elements as expected. In particular, the Au 4f, Al 2p, Au 4d, and O 1s elements are all presented in the XPS survey spectra of PMDM hybrid nanostructures. As shown in the high-resolution XPS spectrum of Au 4f (Figure 2c), two characteristic peaks located at binding energies 84.2 and 87.9 eV are attributed to 4f_7/2_ and 4f_5/2_, respectively, indicating the presence of a metallic state of Au [38]. In addition, the high-resolution XPS analysis (Figure 2d) depicts the peak at 74.1 eV assigned to Al 2p, originating from the Al_2_O_3_ film [39]. The above evidence reveals the existence of dielectric spacer Al_2_O_3_ in the PMDM hybrid nanostructures.

**Figure 3:**
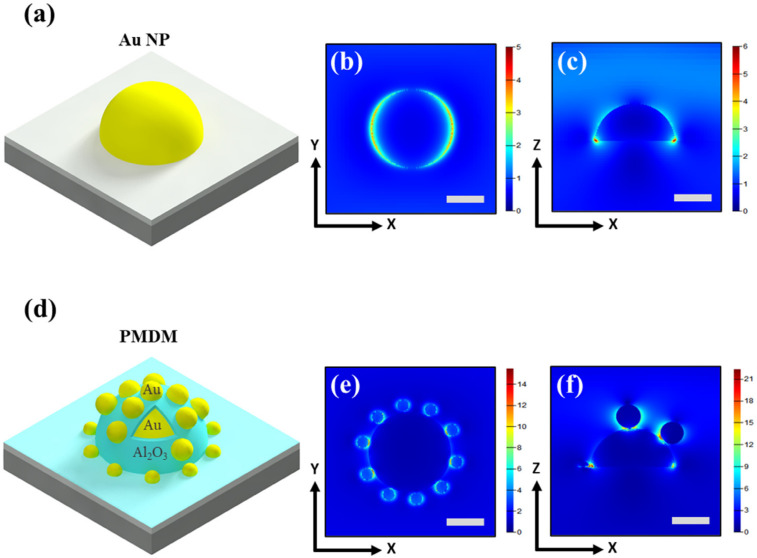
Finite-difference time-domain (FDTD) simulation of EM field distribution of plasmonic nanostructures under the radiation of a 532 nm laser. (**a**) Simulation model and EM field distribution of Au NP at (**b**) X-Y and (**c**) X-Z view. (**d**) Simulation model of PMDM hybrid nanostructure and corresponding EM field distribution in (**e**) X-Y and (**f**) X-Z views. Scale bar: 100 nm.

Next, FDTD simulations were used to calculate the spatial distribution of the near-field EM field of the plasmonic Au and PMDM hybrid nanostructures deposited on SiO_2_/Si substrate. The FDTD simulation models were constructed by mimicking the real experimental results of nanostructures obtained from SEM images, as shown in Figure 3a,d. The FDTD simulation of EM field distribution modes for each nanostructure in the X-Y and X-Z directions was analyzed with an incident laser source with a 532 nm wavelength. As shown in Figure 3b,c, the Au NP provides a hot spot at the interface between the Au NPs and SiO_2_ substrate with a maximal EM field strength (|E|/|E_0_|) of 5.8. Figure 3e,f show the FDTD calculation of the localized EM field distribution in PMDM hybrid nanostructures with a 3 nm Al_2_O_3_ nanogap. The high density of the strongest hot spots is induced at the dielectric Al_2_O_3_ nanogap between the core Au NPs and the out-layered Au NPs due to the plasmon coupling between the Au NPs. Compared with Au NPs, the EM field strength was much higher for the PMDM hybrid nanostructures—i.e., |E|/|E_0_| ≈ 21.5. It is widely known that the SERS EF can be theoretically predicted from the local EM field enhancement (|E|/|E_0_|) of nanostructures—i.e., SERS EF is proportional to the fourth power of |E|/|E_0_| [40,41,42]. Based on the above relation, the theoretical SERS EF is estimated to be ~2.14 × 10^5^ for hybrid nanostructures, which is two orders higher than Au NPs. These results suggest that a high density of strong EM hotspots can be highly beneficial for SERS enhancement. It should be noted that the theoretical calculation of SERS EF from the EM enhancement is usually 2–3 orders lower than the experimental results due to the exclusion of chemical enhancement.

**Figure 4:**
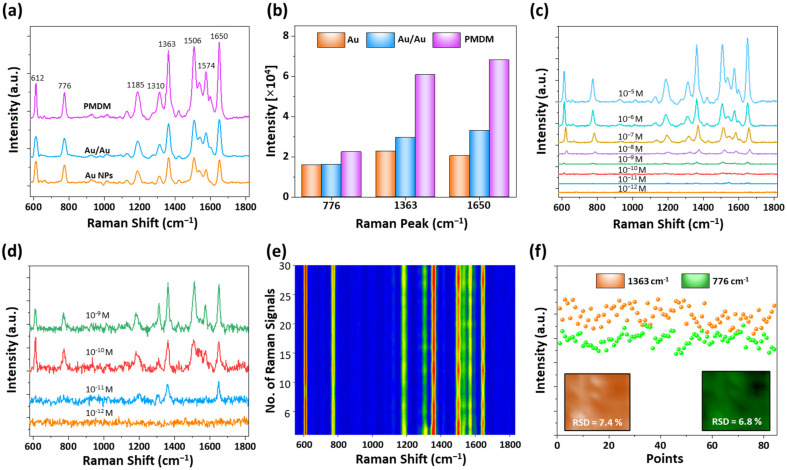
(**a**) Comparison of the SERS spectra of R6G molecules (10^−6^ M) on Au, Au/Au, and PMDM hybrid nanostructure-based SERS substrates. (**b**) Corresponding plot of intensity at Raman peaks of 776, 1363, and 1650 cm^−1^. (**c**) SERS spectra of R6G molecules on PMDM hybrid nanostructure-based SERS substrate with different concentrations ranging from 10^−5^ to 10^−12^ M. (**d**) Magnified SERS spectra of R6G with low concentrations showing the distinct Raman peaks. SERS uniformity and reproducibility of the PMDM hybrid nanostructure substrate. (**e**) SERS contour maps of 30 spots and (**f**) plot of Raman intensities of 776 and 1363 cm^−1^ randomly selected from SERS mapping in an area of 10 μm × 10 μm. (Insets) SERS intensity mapping of 776 and 1363 cm^−1^.

Figure 4 shows the SERS performance of PMDM hybrid nanostructures using R6G as a probe molecule and an excitation laser of wavelength 532 nm. The comparison of the SERS performance of different SERS-active substrates with a 10^−6^ M R6G concentration is demonstrated in Figure 4a, and the corresponding SERS enhancement is summarized in terms of Raman peak intensity in Figure 4b. Several of the most prominent Raman peaks of R6G are observed at the wavenumbers of 612, 776, 1185, 1310, 1363, 1506, 1574, and 1650 cm^−1^, which are consistent with the characteristic peaks of R6G reported in the literature [43,44]. The band assignment of all the Raman peaks of R6G is also summarized in Table S1. In particular, the Raman peak intensity of the PMDM hybrid nanostructures at the wavenumber of 1650 cm^−1^ is about 3.3 and 2.2 times higher compared to that of the Au NPs and Au/Au NPs. As confirmed by the FDTD simulation results, it is obvious that the PMDM hybrid nanostructures exhibit the best performance due to the strong EM field enhancement. It is observed that the intensity of the R6G Raman signal on the hybrid nanostructure is much enhanced, as compared to that of Au NPs. Therefore, the PMDM hybrid nanostructures-based SERS substrates was further analyzed to identify the detection limit, enhancement factor, and reproducibility. Figure 4c,d show the SERS spectra of different concentrations of R6G molecules adsorbed on hybrid nanostructures in the range of 10^−5^ to 10^−12^ M. The Raman intensity is gradually reduced with the decreased R6G concentration. The lowest detectable concentration reaches 10^−11^ M, where certain Raman peaks such as 1363 and 1650 cm^−1^ can be identified, indicating that the SERS substrate possesses a high SERS sensitivity. To quantitatively study the SERS performance of hybrid nanostructures, the SERS EF was calculated using the relation EF = (*I_SERS_*/*C_SERS_*)/(*I_R_*/*C_R_*), where *I_SERS_* and *I_R_* correspond to the Raman peak intensities of R6G obtained from the SERS substrate and reference (SiO_2_) substrate, whereas *C_SERS_* and *C_R_* represent the concentrations of R6G molecules on SERS substrate and reference substrate, respectively. The minimum detectable limit for the SERS substrate was 10^−11^ M, whereas the lowest detection for reference substrate was 10^−2^ M. Therefore, the SERS EF of PMDM hybrid nanostructures for Raman peak 1650 cm^−1^ was estimated as 1.3 × 10^8^, which was much higher than that of the other SERS substrates reported in the literature (Table S2). Furthermore, we tested the reproducibility of the as-prepared SERS substrate by conducting the SERS measurement in several locations. The color contour plot of the SERS spectra of 10^−6^ M R6G measured at random 30 different locations is presented in Figure 4e and the corresponding SERS spectra are presented in Figure S2. The contour plot demonstrates the similar color of Raman signals, signifying the comparable intensity of the Raman signals due to the homogeneous distribution of EM hot spots. In addition, the SERS mapping was performed in an area of 10 μm × 10 μm to further confirm the reproducibility. The RSD values corresponding to Raman peaks 776 and 1363 cm^−1^ were calculated to be 6.8% and 7.4%, respectively, indicating the good reproducibility of SERS substrates. 

## 4. Conclusions

In summary, we developed a facile strategy for a highly sensitive and reproducible SERS substrate based on a hybrid nanostructure. A simple repeated dewetting process coupled with an ALD method was used to fabricate hierarchically assembled PMDM hybrid nano-architectures, which consist of core Au NPs and small out-layered Au NPs isolated by an Al_2_O_3_ layer. FDTD simulation data reveal that the use of the hybrid nanostructures leads to a high density and more intense EM hot spots through the creation of nanogaps by a dielectric spacer. Consequently, the SERS measurements of hybrids nanostructures demonstrate a maximum EF of 1.3 × 10^8^, a low detection limit of 10^−11^ M R6G molecules, and an excellent reproducibility (RSDs less than 7.5%). Thus, we believe that hybrid SERS substrates have the potential to be used in practical applications for the highly sensitive, rapid, and reproducible detection of biomolecules.

## Data Availability

Data are contained within the article or Supplementary Material.

## Supplementary Materials

The following supporting information can be downloaded at: https://www.mdpi.com/article/10.3390/nano12030401/s1. Figure S1: Histogram of Au NPs arrays on Si/SiO_2_ substrate by the annealing of 10 nm Au at 800 °C for 120 s; Figure S2: Raman spectra of R6G molecules (10−6 M) measured at 30 different locations on PMDM hybrid nanostructures to test the SERS reproducibility; Table S1: Raman band assignments of R6G molecules; Table S2: Comparison of SERS performance of current work and previously reported plasmonic NP-based SERS substrates (references [45,46,47,48,49,50,51,52,53] are cited in Table S2).
