# Supplementary material for: Hierarchically Assembled Plasmonic Metal-Dielectric-Metal Hybrid Nano-Architectures for High-Sensitivity SERS Detection
Source: Nanomaterials (Basel). 2022 Jan 26;12(3):401. doi: 10.3390/nano12030401 (PMC8838151; doi:10.3390/nano12030401)
Supplement: Supplementary file 1 [file nanomaterials-12-00401-s001.zip › nanomaterials-1564432-supplementary.pdf]

Supplementary Material

Hierarchically Assembled Plasmonic Metal-Dielectric-Metal Hybrid Nano-Architectures for High-Sensitivity SERS Detection

Puran Pandey[1], Min-Kyu Seo[1], Ki Hoon Shin[1], Young-Woo Lee[2,*] and Jung Inn Sohn[1,*]

[1] Division of Physics and Semiconductor Science, Dongguk University-Seoul, Seoul 04620 Republic of Korea; ppcpurans@gmail.com (P.P.); seominkyuu@gmail.com (M.-K.S.); kihoonshin@dongguk.edu (K.H.S.)

[2] Department of Energy Systems, Soonchunhyang University, Asan-si, Chungcheongnam-do 31538, Republic of Korea

* Correspondence: ywlee@sch.ac.kr (Y.-W.L.); junginn.sohn@dongguk.edu (J.I.S.)

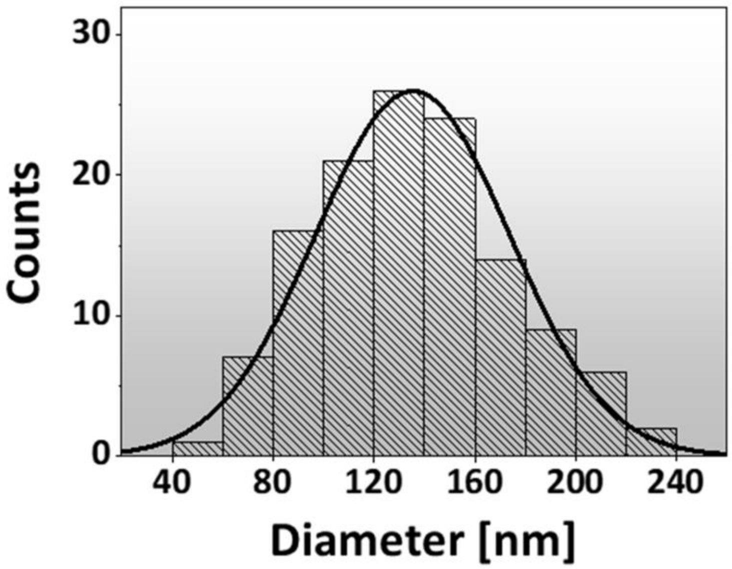

Figure S1. Histogram of Au NPs arrays on Si/SiO_2 substrate by the annealing of 10 nm Au at 800 ^oC for 120 s.

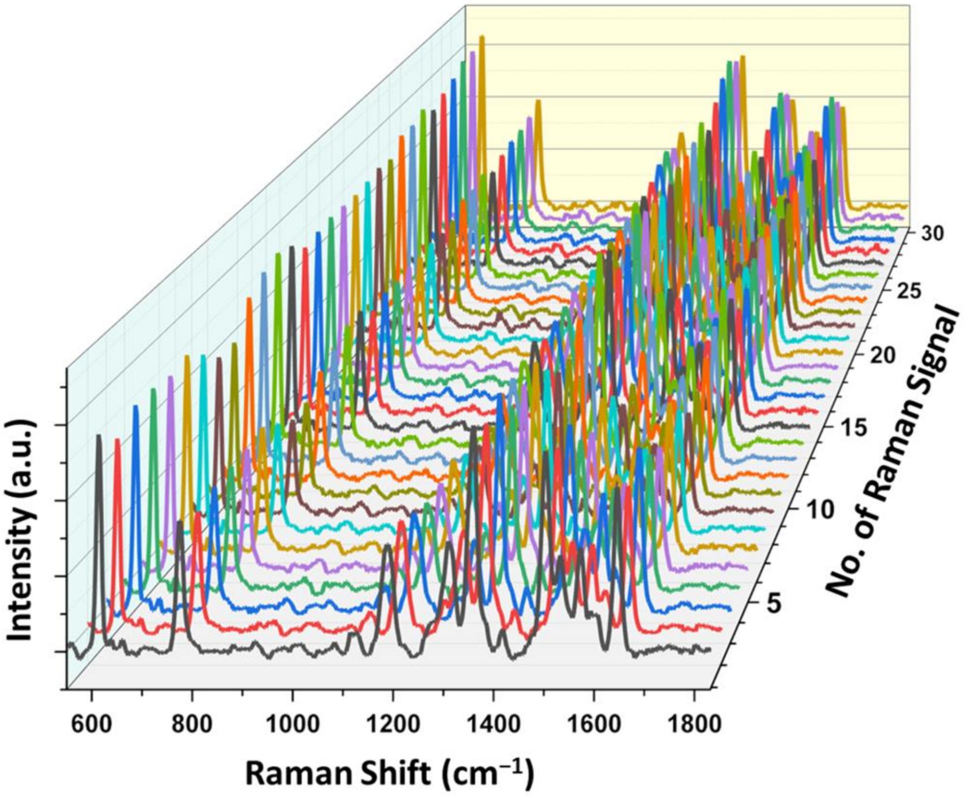

Figure S2. Raman spectra of R6G molecules (10^{-6} M) measured at 30 different locations on PMDM hybrid nanostructures to test the SERS reproducibility.

Table S1. Raman Band Assignments of R6G molecules.

Raman peak [cm^{-1}]		Assignment
**This work**	**Literature** Ref. [45]	
612	612	C−C ring in-plane bending in xanthene/phenyl rings
776	772	C−H out-of-plane bending
1128	1127	C−H in-plane bending in xanthene/phenyl rings
1185	1187	C−H in-plane bending in xanthene ring
1310	1312	hybrid mode (xanthene/phenyl rings and NHC_2H_5 group)
1363	1363	C−C stretching in xanthene ring
1506	1509	C−C stretching in xanthene ring
1575	1575	C−C stretching in phenyl ring
1650	1651	C−C stretching in xanthene ring

Table S2. Comparison of SERS performance of current work and previously reported plasmonic NPs-based SERS substrates.

SERS material	Probe Molecule	LOD	EF	Reference
Au island over Nanosphere	R6G	-	1.5×10^6	[46]
Nanoporous Au-Ag nanorod	4-MBA	10^{-9} M	1.5×10^6	[47]
Nanoporous Au thin films	R6G	10^{-8} M	-	[48]
Au@AuAg multishell Nanostructures	4-ABT	10^{-5} M	9.18×10^5	[49]
Porous AuAg NPs	R6G	2.37×10^{-9} M	7.8×10^6	[4]
Ag@ZrO_2	R6G	10^{-8} M	-	[50]
Sandwich-Like Sensor (AuA-pMIP)	R6G	10^{-10} M	1.24×10^6	[51]
Au/SiO_2/Graphene/Au	CV	-	0.23×10^6	[29]
Ag@SiO_2	R6G	-	1.07×10^7	[52]
Ag/Al_2O_3/Au Nanograting	p-thiocresol	10^{-9} M	5.2×10^7	[53]
Ag-Al_2O_3-Ag heterojunctions	MB	10^{-10} M	1×10^8	[30]
PMDM hybrid nanostructure	R6G	10^{-11} M	1.3×10^8	[This work]

LOD = Limit of Detection
EF = Enhancement Factor
CV = Crystal violet
MB = methylene blue
R6G = Rhodamine 6G
4-MBA = 4-Mercaptobenzoic acid
4-ABT = 4-Aminobenzenethiol
